# Breast Cancer Vaccines: New Insights

**DOI:** 10.3389/fendo.2017.00270

**Published:** 2017-10-13

**Authors:** Rosaria Benedetti, Carmela Dell’Aversana, Cristina Giorgio, Roberta Astorri, Lucia Altucci

**Affiliations:** ^1^Dipartimento di Biochimica Biofisica e Patologia generale, Università degli Studi della Campania ‘L. Vanvitelli’ Naples, Naples, Italy; ^2^Dipartimento di Medicina e Scienze della Salute “Vincenzo Tiberio”, Università degli Studi del Molise, Campobasso, Italy

**Keywords:** breast cancer, vaccines, NeuVax, AVX901, INO-1400

## Abstract

Breast cancer (BC) is a persistent global challenge for its high frequency in women (although it seldom occurs in men), due to the large diffusion of risk factors and gene mutations, and for its peculiar biology and microenvironment. To date, BC can benefit from different therapeutic strategies involving surgery, ablation, chemotherapy, radiotherapy, and more specific approaches such as hormone therapy and the administration of various substances impairing cancer growth, aggressivity, and recurrence with different modalities. Despite these relatively wide chances, also used in combinatory protocols, relevant mortality and relapse rates, often associated with resistant phenotypes, stress the need for a personalized-medicine based on prompting the patient’s immune system (IS) against cancer cells. BC immunogenicity was latterly proven, so the whole immunotherapy field for BC is still at a very early stage. This immunotherapeutic approach exploits both the high specificity of adaptive immune response and the immunological memory. This review is focused on some of the majorly relevant BC vaccines available (NeuVax, AVX901, and INO-1400), providing a description of the more promising clinical trials. The efficacy of cancer vaccines highly depends on the patient’s IS, and a wide optimization is needed in terms of targets’ selection, drug design and combinations, dose finding, protocol structuring, and patients’ recruitment; moreover, new standards are being discussed for the outcome evaluation. However, early-phases excellent results suggest that the manipulation of the IS *via* specific vaccines is a highly attractive approach for BC.

## Introduction

Breast cancer (BC) is the most frequent type of cancer in women worldwide as it represents about 25% of all female cancers though it also unfrequently occurs in men (<1%) (source: World Cancer Research Fund International). Regardless of the decreased mortality rate, due to its high incidence it occupies the fifth position in cancer mortality statistics in women, thus being a significant burden to society and public health. Currently, BC treatment involves multiple strategies such as surgery, ablation, chemotherapy (CT), radiotherapy (RT), hormone therapy (HT), and the use of crucially interfering molecules, antibodies, and antigen/adjuvants to undermine tumor cell growth, proliferation, survival, and invasiveness. Certain therapies can also be used with cytoreductive intent (neoadjuvant). The choice of a therapeutic scheme, suggested by Clinical Practise Guidelines, is related to the existence of BC molecular/immunohistochemical (IHC) subtypes: Luminal A, Luminal B, human epidermal growth factor receptor 2 (HER2)/neu enriched, basal-like and triple-negative breast cancer (TNBC) (1). These subtypes differ for the expression levels of estrogen receptors (ER), progesterone receptor (PR) and HER2/neu, now recognized as tumor-associated antigens. TNBC is instead defined by the lack of ER, PR, and HER2 surface expression, and it is often characterized by a poor prognosis as the only option is CT, with very low response rates (2). However, the majority of BC is constituted by ER^+^ subtypes that can be treated with selective estrogen receptor modulators and selective estrogen receptor downregulators, both reducing the proliferative prompt of ER signaling (3). For HER2/neu-overexpressing BC, therapies based on the use of antibodies against membrane protein targets are available and effective (4, 5). The identification of TAAs is not sufficient to individuate the patients that can be possible good candidates for a specific therapeutic regime, because BC is characterized by a significant resistance to treatments (6). Primary resistance refers to an intrinsic BC property before any exogenous interference (i.e., mutated receptor forms and particular tumor microenvironment) leading to cancer unresponsiveness or partial sensitivity to a specific treatment. Secondary-acquired resistance is instead observed after initial cycles of therapy, and it constitutes an adaptive escape mechanism to therapy-based interference whose most common examples are increased concentrations of steroid receptors and alterations in posttranslational modification patterns, hypoxic phenotypical shifts, and abnormal activation of pathways involving mTOR and/or PI3K. Secondarily resistant phenotypes are a severe dynamical challenge for metastatic BC (MBC), which often ends up into experimental therapy or mere palliative care to improve life quality without giving patients a massive change in life expectancy. Overcoming resistance development has become a priority for the scientific community and several clinical trials are now combining traditional and more recent strategies such as anti-HER2 drugs. Among the most widely used approaches, mTOR and PI3K inhibitors are used in synergy with tamoxifen, fulvestrant, trastuzumab, and lapatinib; more recent protocols also involve epigenetic drugs to interfere with the transcriptional regulation of BC signaling [i.e., human leukocyte antigen (HLA)-G is a non-classical MHC I molecule that protects the fetus from the mother’s IS but it is pathologically expressed in cancer; its levels can be lowered with a combinational strategy involving anti-HLA-G immunotherapy and specific DNA methyltransferase inhibitors] (7). Even in the patients achieving significant response to the combined treatments, the relevant relapse rate highlights the dramatic need for an effective and personalized therapy for BC. A possible solution can rely in the emerging research branch of precision medicine, aiming to a tailored-made approach that appears to be suitable for BC treatment (8). In that case, applied immunotherapy would be strictly related to the single patient status and specific cancer features. There are three different stages during BC progression and maintenance in which the immune system (IS) could be overcome: removal, balance, and escape from canonical surveillance mechanisms. Immunotherapy primarily aims to prompt the removal of tumor cells by increasing both proliferation and activity of T-lymphocytes, antigen presentation, and production of soluble mediators of inflammation (such as cytokines and chemokines) (9). A second specific goal of immunotherapy is to reduce the metastatic potential of BC reducing relapsing rates and resistant secondary tumors development. BC was ultimately confirmed to be an immunogenic tumor (even if not as evidently as other cancer types) so this resulted in a delayed debut of BC immunotherapy (10, 11). For now, it is clear that mammary tumors are infiltrated with immune cells (in particular T cells), and that the amount of infiltrating T-lymphocytes (TILs) is correlated with a better prognosis [Tregs represent an exception to this rule (12)]. Moreover, the activation of TILs is impaired by several tumor microenvironmental factors, such as cytokine levels and tryptophan availability. Both characteristics suggest using specific immune stimuli to engage T-cell proliferation and activation. The existence of well-defined BC TAAs provides the rationale to use specific immunotherapies aimed to push the patient’s IS to selectively attack cancer cells. BC immunotherapies under evaluation for efficacy and safety are as follows: (i) blockade of immunological checkpoints, (ii) T cell-based therapies (autologous or allogenic transfer and/or stimulation), and (iii) vaccinology. The blockade of immune checkpoints mainly consists in the use of inhibitory molecules or antibodies directed against surface immune molecules (receptors or ligands) impairing T-cell proliferation and infiltration (13). Several clinical trials for BC are now evaluating the possibility to target CTLA-4, PD-1, and LAG-3. On the other hand, T-cell stimulation aims to induce the activity of both T-helper and natural killer T cells. Strategies to increase Th1- (CD4^+^) and cytotoxic T-lymphocytes- (CTLs, CD8^+^) dependent immunity and to suppress Th1–Treg joined responses are currently being tested (14). This review will focus on the new perspectives for the use of BC vaccines (15, 16), considering the ongoing clinical trials and the possible future perspectives to combine immunomodulation with other BC therapies. The majority of BC vaccines includes the use of specific BC cellular antigens that serve as targets for the host’s IS. Ideally, this kind of approach could result useful in the primary immune response (*via* amplification mechanisms) and could also lead to develop an adaptive immune surveillance, limiting both the phenomena of metastasis and relapse.

## BC Vaccine

Sophisticated molecular mechanisms orchestrate the immune homeostasis and immune cellular specialization (17). Disturbance of immunological events impacts on cancer development, including BC (18, 19). An intimate crosstalk between BC and the host’s IS occurs in cancer progression, establishing an immune-evasive phenotype and creating an immune suppressive microenvironment (20).

**Figure 1:**
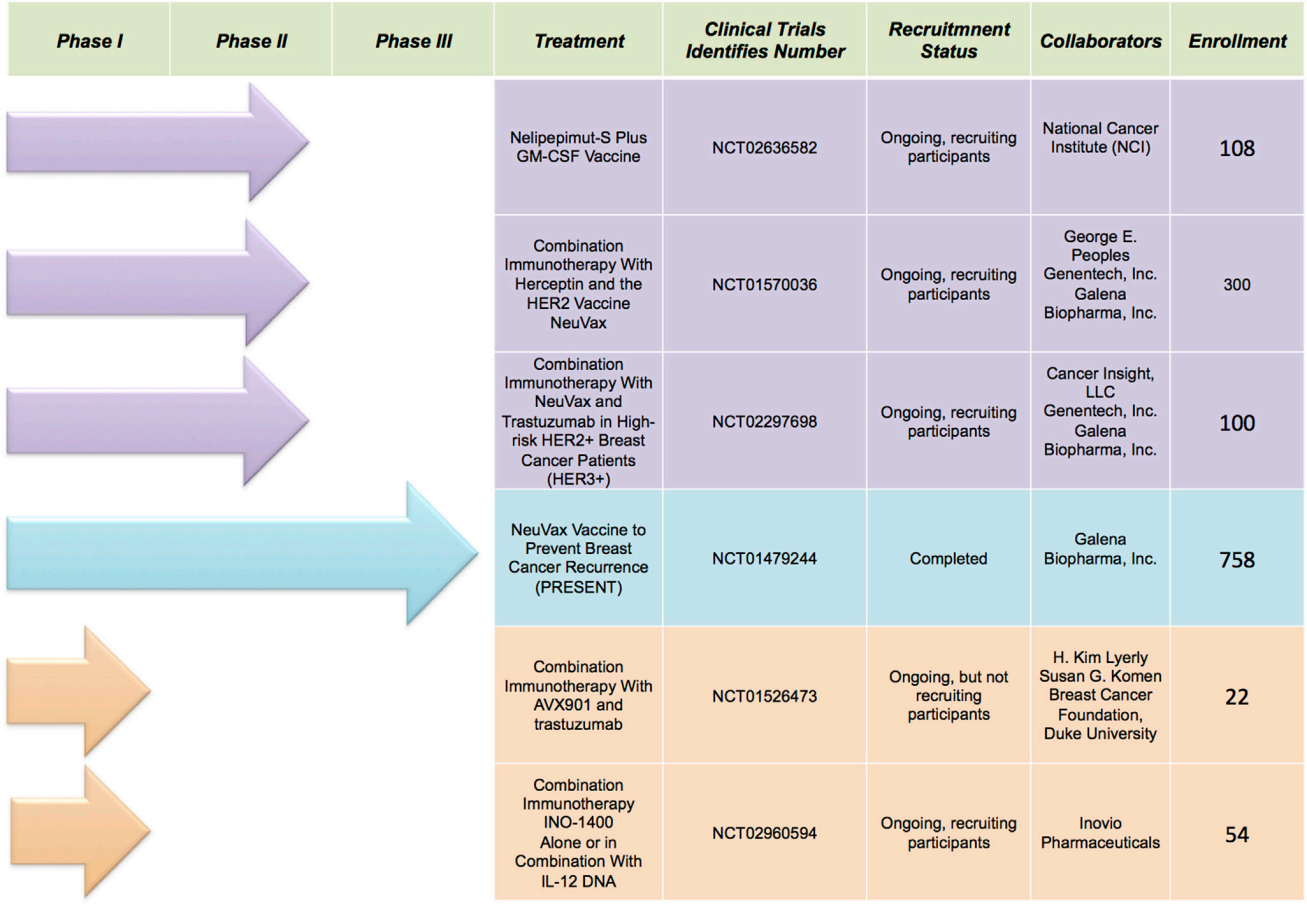
Selected clinical trials discussed in the text (source: http://clinicaltrials.gov).

**Figure 2:**
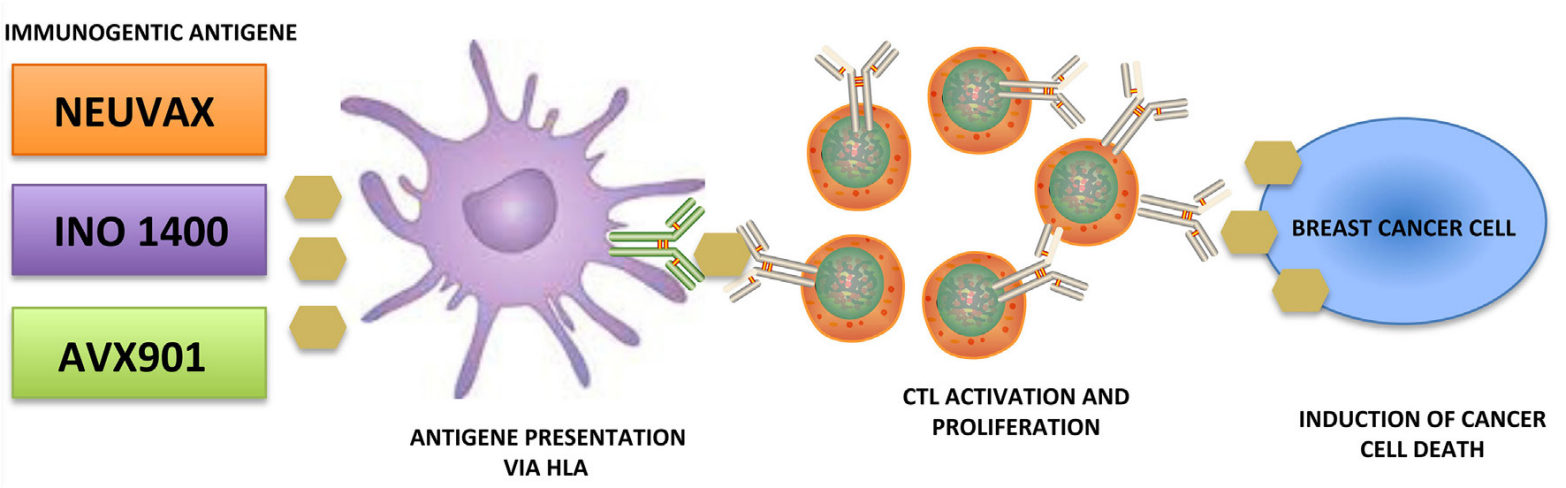
Schematic representation of breast cancer vaccines activity.

Immunotherapy is the new avant-garde in BC therapy. Most promising results are obtained by cancer vaccinology. This immunotherapeutic approach exploits both the high specificity of adaptive immune response and the immunological memory. The clinical potential of BC vaccines consists in its ability to destroy the tumor cells with minimal toxicity. Many different types of cancer vaccines have been constructed from distinct immunogenic sources represented by whole tumor lysates, tumor antigenic peptides, DNA, RNA, and viruses. Moreover, they can be combined with immunoadjuvants, which contribute to the immune stimulation. Encouraging results are coming out during several clinical trials, listed in Figure 1, employing distinct tumor targets and strategies. In this review, we will focus on the most promising BC vaccines currently available: NeuVax, AVX901, and INO-1400 (Figure 2).

### NeuVax

Human epidermal growth factor receptor 2 is the most important TAA functionally implicated in BC pathogenesis (21). HER2 is a tyrosine kinase transmembrane (TM) receptor, which triggers a cascade of downstream signaling leading to proliferation, survival, angiogenesis, and invasion in BC. Overexpression or amplification of HER2 occurs in approximately 15–30% of BC and is associated with shorter disease-free and overall survival in BC. To date, HER2 remains a major target of BC immunotherapy. The current treatment of HER2-positive BC primarily consists in the well known and clinically efficient monoclonal antibodies trastuzumab, Trastuzumab-DM1 (TDM1-Herceptin^®^) and pertuzumab (Perjeta^®^). These represent passive immunotherapy forms, consisting in the administration of immunoglobulins from antigen-specific T-lymphocytes expanded *ex vivo* (22, 23). A new therapeutic approach aims to elicitate an active immunological response through vaccines. Many investigations have examined different immunogenic peptides derived from HER2 and showed differences in the specific immune responses for each (24–26). NeuVax is the most extensively studied BC vaccine against HER2 protein. Its composition comprehends HER2-derived peptide E75 (nelipepimut-S) combined with the immune-adjuvant granulocyte macrophage colony-stimulating factor (GM-CSF). An important advantage of NeuVax consists in the stimulation and the activation of CD8^+^ CTLs and CD8^+^ memory cells against E75, MHC class I epitope. More precisely, the activated specific CTLs bind to HLA-A2/A3 molecules on antigen-presenting cells therefore recognizing, neutralizing and destroying HER2-expressing primary and metastatic cancer cells through cell lysis (27, 28). Initial preclinical studies were conducted to examine the immunogenicity of the E75 peptide (29–32). Subsequently, Phase I trials enrolled patients with MBC with different degrees of HER2 expression (1–3^+^ by IHC), optimizing both doses and inoculation modalities. The study showed that E75 plus GM-CSF administration is acceptably safe and stimulates effective expansion of E75-specific CTLs (33). Since E75 is HLA-restricted, HLA-A2- or HLA-A3-positive patients were vaccinated, whereas HLA-A2/A3-negative patients were followed prospectively as unvaccinated controls. Patients were administered escalating doses of E75 plus GM-CSF monthly for 4 or 6 months. The vaccination series was well tolerated, with minimal toxicity at all dose levels. These encouraging data were supported with the trials transition into Phase II studies. The results have strongly demonstrated that vaccine therapy administered to hormone receptor-positive patients improves overall survival and is well tolerated, with generally limited toxicities, including erythema and pruritus at the injection site, mild influenza-like symptoms, fatigue, and bone pain. NeuVax is now the only BC vaccine that completed Phase III clinical trial. In February 2017, PRESENT (Prevention of Recurrence in Early-Stage, Node-Positive BC with Low to Intermediate HER2 Expression with NeuVax Treatment) successfully concluded the evaluation of treatment with NeuVax plus GM-CSF versus placebo plus GM-CSF to prevent cancer recurrence in node positive, HER2 IHC 1^+^/2^+^, and HLA-A2^+^ and/or A3^+^ patients (ClinicalTrials.gov identifier: NCT01479244). In addition to PRESENT, the biopharmaceutical company Galena Biopharma developed other two studies with NeuVax in combination with trastuzumab (Herceptin^®^; Genentech/Roche) for BC: a Phase IIb trial in node positive (or node negative if negative for both ER and PR) BC patients with HER2 IHC 1^+^/2^+^ (ClinicalTrials.gov identifier: NCT01570036); a Phase II trial in high-risk node positive or negative HER2 IHC 3^+^ patients (ClinicalTrials.gov identifier: NCT02297698). Moreover, Phase II trial of the Nelipepimut-S Peptide vaccine in women with ductal carcinoma *in situ* (DCIS), aims to assess whether nelipepimut-S plus GM-CSF or sargramostim (Leukine^®^) is more effective in treating patients with DCIS (ClinicalTrials.gov Identifier: NCT02636582). The excellent results suggest that the manipulation of the IS is a highly attractive approach. Development of more innovative vaccines, more potent immune adjuvants and more appropriate ways of administration remains a substantial goal for patients with HER2-positive BC.

### AVX901

An investigational antigen-specific cancer vaccine for BC is represented by virus-like replicon particle (VRP)-HER2 (now called AVX901). It is developed from an attenuated strain of Venezuelan equine encephalitis virus (VEE) showing a potential antineoplastic activity. This vaccine vector system is modified to be non-infectious, with the genes encoding the VEE structural proteins replaced with the extracellular domain and TM regions of HER2 gene, creating a propagation-defective single-cycle self-amplifying RNA (replicon) that highly expresses HER2. In particular, VRP-HER2 ECDTM replicon is packaged into VRPs by providing the full complement of VEE structural proteins from two separate helper RNA molecules (34). Several preclinical studies assessed the extreme effectiveness of VRP-HER2 vaccines to activate cellular and humoral immune responses against HER2, resulting in reduced tumor growth in both orthotopic xenograft mouse models and in human HER2-transgenic mice (35–37). Wolpoe and colleagues reported a significant increase of the antitumor immunity in HER2/neu transgenic mice by combining HER2-targeted vaccination with trastuzumab (38). In detail, after immunization with AVX901, the VRPs are able to exclusively infect the cells into which they are introduced, and the replicon may express large amounts of HER2 protein, directing both activated CTLs and CD8^+^ memory cells against HER2-expressing cancer cells. Furthermore, AVX901 is not only able to address the IS against HER2/neu-overexpressing malignant cells, but also to block the signaling of wild-type HER2, thus limiting its tumor sustaining activity. In a Phase I clinical trial (NCT01526473), AVX901 was tested in 22 patients with HER2-overexpressing progressive or MBC, alone or in conjunction with other HER2-targeted therapies (e.g., trastuzumab, trastuzumab plus pertuzumab, T-DM1, or lapatinib). The primary and the secondary endpoints of this interventional study are to evaluate the safety and to determine the antitumor immune activity, respectively. Early clinical evidences did not report any dose-limiting toxicity, supporting VRP-HER2 safety in humans, but further tests will be performed to monitor the tumor response rate. This research is already underway, but not yet recruiting.

### INO-1400

hTERT is an attractive target recognized by the IS for cancer vaccination. Telomerase is a ribonucleoprotein enzyme that maintains chromosomal length and stability by protecting telomeric DNA, leading to cellular immortalization (39). hTERT is hyper expressed in over 85% of human cancers, including BC (40). The aberrant expression is associated with long-term survival and unlimited proliferation of malignant cells (41). All these findings support the study of potential hTERT vaccines for cancer immunotherapy (42). However, few studies have been using hTERT DNA vaccines in advanced BC (43). In this scenario, synthetic hTERT DNA vaccine INO-1400 has emerged as a novel approach for antigen-specific immunotherapy in BC. It is composed of a plasmid encoding the catalytic subunit of TAA hTERT, with two differentiating immunogenic mutations, eliciting a broad CTL-mediated response against tumor cells, triggering the hTERT antigen. This full-length hTERT DNA vaccine (phTERT) is able to break the immune tolerance and to intensify the killing of hTERT-pulsed target cells. Yan and colleagues demonstrated the potent antitumor immunity of hTERT DNA vaccine in both mice and monkeys in preclinical models. In detail, its administration in mice generated a strong cellular immune response with an increased number of the cells producing CD107a, IFN-γ and TNF-α (44). In addition, the data showed that monkeys immunized with hTERT DNA vaccine exhibited significantly slowed tumor growth and longer overall survival compared with those in the naïve group; no vaccine-induced cytotoxic effects or organ damage were detected (44). Further studies will be required to improve both the setting of immunotherapy and tumor immune surveillance in patients with high risk of relapse. In an ongoing Phase I clinical trial (NCT02960594), INO-1400 will be delivered *via* intradermal injection, alone or in conjunction with Inovio’s IL-12 immune activator (INO-9012) in patients with BC, pancreatic or lung cancer at high risk of relapse after surgery and other standard adjuvant therapy. Over 50 subjects affected by at least one of nine different hTERT-expressing cancers will be enrolled into one of the six treatment arms. This clinical trial is an open-label dose-escalation study with the primary purpose of establishing the safety and tolerability of hTERT DNA vaccine. Finally, the secondary endpoint of the trial will be to determine the connections between immune responses and clinical outcomes.

## Conclusion

The relatively recent epiphanies in cancer immune biology raised new opportunities and challenges in cancer therapy, with the major aim to remodulate the patients’ IS responses against cancer cells. BC is a worldwide critical issue for its high frequency in women (although not exclusively), due to the large prevalence of risk factors and gene mutations, and for its particular biology and microenvironment.

In fact, among the trickier peculiarities of this cancer type there are the surprising variability in receptors’ expression, the differential presence of Treg cells, TILs, tertiary lymphoid structures, and macrophages (12, 45, 46), the role of both senescent elements (47) and cancer stem cells, the relatively poor immunogenicity of certain subtypes. These factors strongly influence the prognosis and the clinical outcome of the treatment. Despite all the difficulties and the delay in comparison with other malignancies (i.e., melanoma, NSCLC, and hematological cancer), BC immunotherapy had a lagging start and a recent burst with a rapidly increasing number of trials. The rational basis of immunotherapy relies in the possibility to target known antigenic molecules differentially or preferentially expressed by tumor cells, the TAAs, using immunotherapeutic strategy alone or in combination with preexistent therapies of large use (i.e., surgery, ablation, CT, RT, and HT) with a possible synergistic effect. An ideal target for immunotherapy should encompass various characteristics such as frequent overexpression in a sufficient percentage of cancer cells (preferentially or exclusively) versus normal cells and a function whose disruption may represent an advantage itself [i.e., the transcription factor, brachyury, a new BC vaccine target (2)]. Multiple omic approaches, immunoinformatics, genetic platforms, high-speed sequencers, and data analysis will be needed (48, 49). On the other hand, cancer vaccines represent a golden chance for the amenable BC subtypes. Polyvalent constructions appeared to elicitate minor resistance development but more toxicity and immune adverse events in comparison with monovalent options (50). As for any other vaccine, cancer vaccines highly depend on the subject’s IS. Both the strategies need a wide optimization in terms of dose finding, patients’ selection, combinatory protocol construction, and technical problem solving to improve target detection, antigen presentation, drug delivery, specific response elicitation in terms of cells and cytokines, response evaluation (in plasma samples or with molecular and traditional imaging with modified criteria RECIST to iRECIST “immune-related Response Evaluation Criteria In Solid Tumors”) and to reduce the risk of adverse events and recidives (51, 52). The huge chapter of the immune adjuvants of endogen or exogen origin has to be deepened and may represent a major area of interest for the future. Immunotherapy is currently being tested for solid and hematological cancer from early to metastatic stage, with protocols involving it with an anxillary role or as a protagonist in cancer fighting for an increasing number of patients, eluding escaping variants (14, 49, 53). To let immunotherapy be the wished revolution in cancer therapy including BC, bioinformatics, drug design/pharmacology, molecular biology, data analysis, clinical science, and medical imaging will need to share an emerging mentality.

## Author Contributions

LA and RB drafted the work and revised it critically for important intellectual content; both the authors finally approved the version to be submitted. RB and CD contributed equally to this paper writing several sections. CG and RA helped to write and read literature.

## Conflict of Interest Statement

The authors declare that the research was conducted in the absence of any commercial or financial relationships that could be construed as a potential conflict of interest. The handling editor declared a shared affiliation, though no other collaboration, with several of the authors LA, RB, CD, and CG.
